# Perturbation of convex risk minimization and its application in differential private learning algorithms

**DOI:** 10.1186/s13660-016-1280-0

**Published:** 2017-01-05

**Authors:** Weilin Nie, Cheng Wang

**Affiliations:** Huizhou University, Huizhou, P.R. China

**Keywords:** differential privacy, convex risk minimization, perturbation, concentration inequality, error decomposition

## Abstract

Convex risk minimization is a commonly used setting in learning theory. In this paper, we firstly give a perturbation analysis for such algorithms, and then we apply this result to differential private learning algorithms. Our analysis needs the objective functions to be strongly convex. This leads to an extension of our previous analysis to the non-differentiable loss functions, when constructing differential private algorithms. Finally, an error analysis is then provided to show the selection for the parameters.

## Introduction

In learning theory, convex optimization is one of the powerful tools in analysis and algorithm designs, which is especially used for empirical risk minimization (ERM) (Vapnik 1998 [1]). When running on a sensitive data set, algorithms may leak private information. This has motivated the notion of differential privacy (Dwork *et al.* 2006, 2016 [2, 3]).

For the sample space *Z*, denote the Hamming distance between two sample sets $\{\mathbf{z}_{\boldsymbol{1}},\mathbf{z}_{\boldsymbol{2}}\} \in {Z}^{m}$ as

$$d({\mathbf{z}_{\boldsymbol{1}},\mathbf{z}_{\boldsymbol{2}}})=\# \{i=1,\ldots,m: z_{1,i} \neq z_{2,i}\}, $$

*i.e.*, there is only one element that is different. Then *ϵ*-differential privacy is defined as follows.

### Definition 1

A random algorithm $A: Z^{m} \to \mathcal{H}$ is *ϵ*-differential private if for every two data sets ${\mathbf{z}_{\boldsymbol{1}}}$, ${\mathbf{z}_{\boldsymbol{2}}}$ satisfying $d({\mathbf{z}_{\boldsymbol{1}},\mathbf{z}_{\boldsymbol{2}}})=1$, and every set $\mathcal{O} \in \operatorname{Range}(A({\mathbf{z}_{\boldsymbol{1}}})) \cap \operatorname{Range}(A({\mathbf{z}_{\boldsymbol{2}}}))$, we have

$$\Pr \bigl\{ A({\mathbf{z}_{\boldsymbol{1}}}) \in \mathcal{O} \bigr\} \leq e^{\epsilon}\cdot \Pr \bigl\{ A({\mathbf{z}_{\boldsymbol{2}}}) \in \mathcal{O} \bigr\} . $$

Throughout the paper, we assume $\epsilon<1$ for meaningful privacy guaranties. The relaxation $(\epsilon, \delta)$-differential privacy is also interesting and has been studied in some recent literature. However, it is out of our scope and we will just focus on the *ϵ*-differential privacy throughout the paper. Extension of our results to $(\epsilon, \delta)$-differential privacy or concentrated differential privacy [3] may be studied in future work.

A mechanism obtains differential privacy usually by adding a perturbation term to an original definite output (Dwork *et al.* 2006 [4]), *i.e.*, the so-called Laplacian mechanism. McSherry and Talwar 2007 [5] proposed the exponential mechanism, which chooses an output based on its utility function. Indeed, the two mechanisms are related, and both of them are dependent with some kinds of sensitivity of the original definite output. We refer to Dwork 2008 [6] and Ji *et al.* 2014 [7] for a general idea of the differential private algorithms and applications.

A line of work, beginning with Chaudhuri *et al.* 2011 [8], introduced the output perturbation and objective perturbation algorithm to obtain differential privacy for the ERM algorithms. This is following [9–13], etc. However, most of the literature needs a differentiable loss function, sometimes a double-differentiable condition is required (see [8] for detail analysis). This limits the application for the algorithms, such as ERM algorithms with hinge loss (SVM) or pinball loss ([14]), and it motivates our work.

On the other hand, sensitivity in a differential private algorithm, which can be considered as the perturbation for the ERM algorithms, or the stability, has been studied in Bousquet and Elisseeff 2002 [15] and Shalev-Shwartz *et al.* 2010 [16] in the classical learning theory setting. More recently, the relationship between the stability and differential privacy has been revealed in Wang *et al.* 2015 [17].

The main contribution of this paper is to present a different perturbation analysis for the ERM algorithms, in which the condition is just in having convex loss functions and strongly convex regularization terms. Thus the output perturbation mechanisms can still be valid directly in SVM or other non-differentiable loss cases. Besides, an error analysis is conducted, from which we find a choice for the parameter *ϵ* to balance the privacy and generalization ability.

## Perturbation analysis for ERM algorithms

In this section we consider the general regularized ERM algorithms. Let *X* be a compact metric space, and output $Y \subset \mathbb{R}$, where $\vert y\vert \leq M$ for some $M>0$. (We refer to Cucker and Smale 2002 [18] and Cucker and Zhou 2007 [19] for more details as regards this learning theory setting.) A function $f_{{\mathbf{z}},\mathcal{A}}: X \to Y$ is obtained via some algorithm $\mathcal{A}$ based on the sample ${\mathbf{z}}=\{z_{i}\}_{i=1}^{m}=\{(x_{i}, y_{i})\}_{i=1}^{m}$, which is drawn according to a distribution function *ρ* on the sample space $Z:=X \times Y$. Furthermore, we assume there is a marginal distribution $\rho_{X}$ on *X* and a conditional distribution $\rho(y\vert x)$ on *Y* given some *x*.

Firstly we introduce our notations which will be used in the following statements and analysis. Let the loss function $L(f(x),y)$ be positive and convex for the first variable. Denote

$$\begin{aligned}& \mathcal{E}(f)= \int_{Z} L\bigl(f(x),y\bigr) \,d\rho, \\& \mathcal{E}_{\mathbf{z}}(f)=\frac{1}{m} \sum _{i=1}^{m} L\bigl(f(x_{i}),y_{i} \bigr). \end{aligned}$$

Without loss of generality, we set $\bar{\mathbf{z}}=\{z_{1},z_{2}, \ldots, z_{m-1},\bar{z}_{m}\}$, which replaces the last element of **z**, and ${\mathbf{z}\boldsymbol{-}}=\{z_{1},z_{2}, \ldots, z_{m-1}\}$ as a sample set deleting the last element of **z**. Then similar notations can be given:

$$\begin{aligned}& \mathcal{E}_{\bar{\mathbf{z}}}(f)=\frac{1}{m} \Biggl( \sum _{i=1}^{m-1} L\bigl(f(x_{i}),y_{i} \bigr) + L\bigl(f(\bar{x}_{m}),\bar{y}_{m}\bigr) \Biggr), \\& \mathcal{E}_{\mathbf{z}\boldsymbol{-}}(f)=\frac{1}{m-1} \sum _{i=1}^{m-1} L\bigl(f(x_{i}),y_{i} \bigr). \end{aligned}$$

Denote $(\mathcal{H}_{K}, \Vert \cdot \Vert _{K})$ as the reproducing kernel Hilbert space (RKHS) on *X*, *i.e.*, $\mathcal{H}_{K}:=\overline{\operatorname{span}\{K(x, \cdot), x \in X\}}$, where $K:X \times X \to \mathbb{R}$ is a Mercer kernel. Let $K_{x}(y)=K(x,y)$ for any $x,y \in X$, and $\kappa=\sup_{x,y \in X} \sqrt{K(x,y)}$. Then the reproducing property tells us that $f(x)=\langle f, K_{x} \rangle_{K}$. Now a typical regularized ERM algorithm can be stated as

1 $$ f_{\mathbf{z}}=\arg\min_{f \in \mathcal{H}_{K}} \frac{1}{m} \sum_{i=1}^{m} L\bigl(f(x_{i}),y_{i} \bigr)+\lambda\Omega(f). $$

Here $\lambda>0$ is the regularization parameter and $\Omega(f)$ is a *γ*-strongly ($\gamma>0$) convex function with respect to the *K* norm, *i.e.*, for any $f_{1}, f_{2} \in \mathcal{H}_{K}$ and $t \in [0,1]$,

$$\Omega\bigl(tf_{1}+(1-t)f_{2}\bigr) \leq t \Omega(f_{1})+(1-t)\Omega(f_{2}) -\frac{\gamma}{2} t(1-t) \Vert f_{1}-f_{2}\Vert _{K}^{2}. $$

This definition of being strongly convex is taken from Sridharan 2008 [20], where the authors derived some kind of uniform convergence under the strongly convex assumption. It has been widely used in the subsequent literature such as [8, 12, 16, 17], etc. By denoting

$$\begin{aligned}& f_{\bar{\mathbf{z}}}=\arg \min_{f \in \mathcal{H}_{K}} \mathcal{E}_{\bar{\mathbf{z}}} (f)+\lambda \Omega(f), \\& f_{\mathbf{z}\boldsymbol{-}}=\arg \min_{f \in \mathcal{H}_{K}} \mathcal{E}_{\mathbf{z}\boldsymbol{-}}(f) +\lambda \Omega(f), \end{aligned}$$

we have the following result.

### Theorem 1

*Let* $f_{\mathbf{z}}$ *and* $f_{\bar{\mathbf{z}}}$ *be defined as above*. Ω *is* *γ*-*strongly convex and* *L* *is convex w*.*r*.*t*. *the first variable*. *Assume there is a* $B>0$ *such that* $\lambda \Omega(f_{S}) \leq B$ *and* $\vert L(f_{S}(x),y)\vert \leq B$ *for any* ${\mathbf{S}} \in Z^{m}$, $m \in \mathbb{N}$ *and* $(x,y) \in Z$. *Then we have*

$$\Vert f_{\mathbf{z}}-f_{\bar{\mathbf{z}}}\Vert _{K} \leq \sqrt{ \frac{16B}{\lambda \gamma m}}. $$

### Proof

We will prove the result in three steps.

(1) For any $S \in Z^{m}$ and $f_{S}$ from (1),

$$\bigl\vert \mathcal{E}_{\mathbf{z}}(f_{S})-\mathcal{E}_{\bar{\mathbf{z}}}(f_{S}) \bigr\vert \leq \frac{2B}{m}. $$

It is obvious from the definition above that

$$\bigl\vert \mathcal{E}_{\mathbf{z}}(f_{\mathbf{S}})-\mathcal{E}_{\bar{\mathbf{z}}}(f_{\mathbf{S}}) \bigr\vert \leq \frac{1}{m} \bigl\vert L\bigl(f_{\mathbf{S}}(x_{m}),y_{m} \bigr)-L\bigl(f_{\mathbf{S}}(\bar{x}_{m}),\bar{y}_{m}\bigr) \bigr\vert \leq \frac{2B}{m}. $$

(2) The minimization of the two objective functions are close, *i.e.*,

$$\bigl\vert \bigl( \mathcal{E}_{\mathbf{z}}(f_{\mathbf{z}})+\lambda \Omega(f_{\mathbf{z}}) \bigr)- \bigl( \mathcal{E}_{\bar{\mathbf{z}}}(f_{\bar{\mathbf{z}}})+ \lambda \Omega(f_{\bar{\mathbf{z}}}) \bigr) \bigr\vert \leq \frac{2B}{m}. $$

From the notations above, we have

$$\mathcal{E}_{\mathbf{z}}(f_{\mathbf{z}\boldsymbol{-}})+\lambda \Omega(f_{\mathbf{z}\boldsymbol{-}}) \geq \mathcal{E}_{\mathbf{z}}(f_{\mathbf{z}})+\lambda \Omega(f_{\mathbf{z}}), $$

*i.e.*,

$$\begin{aligned}& \sum_{i=1}^{m} L\bigl(f_{\mathbf{z}\boldsymbol{-}}(x_{i}),y_{i} \bigr)+\lambda m \Omega(f_{\mathbf{z}\boldsymbol{-}})\\& \quad \geq \sum_{i=1}^{m} L\bigl(f_{\mathbf{z}}(x_{i}),y_{i}\bigr)+\lambda m \Omega(f_{\mathbf{z}}) \\& \quad \geq \sum_{i=1}^{m-1} L \bigl(f_{\mathbf{z}}(x_{i}),y_{i}\bigr)+\lambda(m-1) \Omega(f_{\mathbf{z}}) \geq \sum_{i=1}^{m-1} L\bigl(f_{\mathbf{z}\boldsymbol{-}}(x_{i}),y_{i}\bigr) +\lambda(m-1) \Omega(f_{\mathbf{z}\boldsymbol{-}}). \end{aligned}$$

A similar analysis for $f_{\bar{\mathbf{z}}}$ can be given as follows:

$$\begin{aligned}& \sum_{i=1}^{m-1} L\bigl(f_{\mathbf{z}\boldsymbol{-}}(x_{i}),y_{i} \bigr) + L\bigl(f_{\mathbf{z}\boldsymbol{-}}(\bar{x}_{m}), \bar{y}_{m} \bigr)+\lambda m \Omega(f_{\mathbf{z}\boldsymbol{-}})\\& \quad \geq \sum_{i=1}^{m-1} L\bigl(f_{\bar{\mathbf{z}}}(x_{i}),y_{i}\bigr) + L \bigl(f_{\bar{\mathbf{z}}}(\bar{x}_{m}),\bar{y}_{m}\bigr)+\lambda m \Omega(f_{\bar{\mathbf{z}}}) \\& \quad \geq \sum_{i=1}^{m-1} L \bigl(f_{\bar{\mathbf{z}}}(x_{i}),y_{i}\bigr) +\lambda(m-1) \Omega(f_{\bar{\mathbf{z}}}) \geq \sum_{i=1}^{m-1} L\bigl(f_{\mathbf{z}\boldsymbol{-}}(x_{i}),y_{i}\bigr) +\lambda(m-1) \Omega(f_{\mathbf{z}\boldsymbol{-}}). \end{aligned}$$

Note that $\sum_{i=1}^{m} L(f_{\mathbf{z}}(x_{i}),y_{i})+\lambda m \Omega(f_{\mathbf{z}})$ is indeed $m (\mathcal{E}_{\mathbf{z}}(f_{\mathbf{z}})+\lambda \Omega(f_{\mathbf{z}}) )$, and the two lower bounds above is the same, we have

$$\begin{aligned}& \bigl\vert m \bigl[ \bigl( \mathcal{E}_{\mathbf{z}}(f_{\mathbf{z}})+\lambda \Omega(f_{\mathbf{z}}) \bigr) - \bigl( \mathcal{E}_{\bar{\mathbf{z}}}(f_{\mathbf{z}})+ \lambda \Omega(f_{\bar{\mathbf{z}}}) \bigr) \bigr] \bigr\vert \\& \quad \leq \max \bigl\{ L\bigl(f_{\mathbf{z}\boldsymbol{-}}(x_{m}),y_{m} \bigr)+\lambda\Omega(f_{\mathbf{z}\boldsymbol{-}}), L\bigl(f_{\mathbf{z}\boldsymbol{-}}( \bar{x}_{m}),\bar{y}_{m}\bigr)+\lambda\Omega(f_{\mathbf{z}\boldsymbol{-}}) \bigr\} . \end{aligned}$$

We can deduce that

$$\bigl\vert \bigl( \mathcal{E}_{\mathbf{z}}(f_{\mathbf{z}})+\lambda \Omega(f_{\mathbf{z}}) \bigr)- \bigl( \mathcal{E}_{\bar{\mathbf{z}}}(f_{\bar{\mathbf{z}}})+ \lambda \Omega(f_{\bar{\mathbf{z}}}) \bigr) \bigr\vert \leq \frac{2B}{m}. $$

(3) Now we can prove our main result. Since Ω is *γ*-strongly convex, and $L(f(x),y)$ is convex w.r.t. the first argument, which leads to the convexity of $\mathcal{E}_{\mathbf{z}}(f)$, for any $0< t<1$, it follows that

$$\begin{aligned}& \mathcal{E}_{\mathbf{z}}(f_{\mathbf{z}}) +\lambda \Omega(f_{\mathbf{z}})\\& \quad \leq \mathcal{E}_{\mathbf{z}}\bigl(tf_{\mathbf{z}}+(1-t)f_{\bar{\mathbf{z}}}\bigr) +\lambda \Omega\bigl(tf_{\mathbf{z}}+(1-t)f_{\bar{\mathbf{z}}}\bigr) \\& \quad \leq t\mathcal{E}_{\mathbf{z}}(f_{\mathbf{z}})+(1-t) \mathcal{E}_{\mathbf{z}} (f_{\bar{\mathbf{z}}}) +\lambda \biggl[ t \Omega(f_{\mathbf{z}})+(1-t) \Omega(f_{\bar{\mathbf{z}}})-\frac{\gamma}{2} t(1-t) \Vert f_{\mathbf{z}} -f_{\bar{\mathbf{z}}}\Vert ^{2}_{K} \biggr] \\& \quad =t \bigl(\mathcal{E}_{\mathbf{z}}(f_{\mathbf{z}})+\lambda \Omega(f_{\mathbf{z}}) \bigr) +(1-t) \bigl( \mathcal{E}_{\mathbf{z}}(f_{\bar{\mathbf{z}}})+ \lambda \Omega(f_{\bar{\mathbf{z}}}) \bigr)-\frac{\lambda\gamma}{2} t(1-t)\Vert f_{\mathbf{z}} -f_{\bar{\mathbf{z}}}\Vert ^{2}_{K} \\& \quad \mathop{\leq}^{(1)} t \bigl(\mathcal{E}_{\mathbf{z}}(f_{\mathbf{z}}) +\lambda\Omega(f_{\mathbf{z}}) \bigr) +(1-t) \biggl( \mathcal{E}_{\bar{\mathbf{z}}} (f_{\bar{\mathbf{z}}})+\lambda \Omega(f_{\bar{\mathbf{z}}}) +\frac{2B}{m} \biggr) - \frac{\lambda\gamma}{2} t(1-t)\Vert f_{\mathbf{z}} -f_{\bar{\mathbf{z}}}\Vert ^{2}_{K} \\& \quad \mathop{\leq}^{(2)} t \bigl(\mathcal{E}_{\mathbf{z}}(f_{\mathbf{z}}) +\lambda\Omega(f_{\mathbf{z}}) \bigr) +(1-t) \biggl( \mathcal{E}_{\mathbf{z}} (f_{\mathbf{z}})+\lambda \Omega(f_{\mathbf{z}}) +\frac{4B}{m} \biggr) - \frac{\lambda\gamma}{2} t(1-t)\Vert f_{\mathbf{z}} -f_{\bar{\mathbf{z}}}\Vert ^{2}_{K} \\& \quad =\mathcal{E}_{\mathbf{z}}(f_{\mathbf{z}}) +\lambda \Omega(f_{\mathbf{z}}) +\frac{4(1-t)B}{m}-\frac{\lambda\gamma}{2} t(1-t)\Vert f_{\mathbf{z}} -f_{\bar{\mathbf{z}}}\Vert ^{2}_{K}. \end{aligned}$$

Therefore,

$$\frac{\lambda\gamma t}{2}\Vert f_{\mathbf{z}}-f_{\bar{\mathbf{z}}}\Vert ^{2}_{K} \leq \frac{4B}{m}. $$

Simply taking $t=\frac{1}{2}$ we have

$$\Vert f_{\mathbf{z}}-f_{\bar{\mathbf{z}}}\Vert _{K} \leq \sqrt{ \frac{16B}{\lambda \gamma m}}, $$

which proves our result. □

Now let us make a brief remark about this result. In our theorem, only convexity for the loss function and *γ*-strongly convexity for Ω are assumed. The assumption $\lambda\Omega(f_{\mathbf{S}}) \leq B$ is trivial for algorithms such as general SVM or coefficient regularization [21], since $\mathcal{E}_{\mathbf{S}}(f_{\mathbf{S}})+\lambda\Omega(f_{\mathbf{S}})$ is the minimum value. The advantage of this result is that most of our learning algorithms satisfy this condition, especially including hinge loss for SVM and pinball loss for quantile regression. Perturbation, or stability analysis has already been performed in [15, 16]. There the authors proposed quite a few stability definitions, which is mainly used for classical generalization analysis. References [10, 22] also studied the differential private learning algorithms with different kernels and Lipschitz losses, with a regularization term of square norm. A similar result to theirs with our notations is as follows.

### Theorem 2

*Let* $f_{\mathbf{z}}$, $f_{\bar{\mathbf{z}}}$, $f_{\mathbf{z}\boldsymbol{-}}$ *be defined as above*. *Assume* $\vert L(t_{1},y)-L(t_{2},y)\vert \leq C_{L}\vert t_{1}-t_{2}\vert $ *for any* $t_{1}$, $t_{2}$, *y* *and some* $C_{L}>0$, *then we have*

$$\Vert f_{\mathbf{z}}-f_{\bar{\mathbf{z}}}\Vert _{K} \leq \frac{2\kappa C_{L}}{\lambda \gamma m}. $$

### Proof

From the convexity of the loss function and regularization term, we have, for any $f \in \mathcal{H}_{K}$ and $0< t<1$,

$$\begin{aligned} \mathcal{E}_{\mathbf{z}}(f_{\mathbf{z}})+\lambda \Omega(f_{\mathbf{z}}) &\leq \mathcal{E}_{\mathbf{z}}\bigl(tf_{\mathbf{z}}+(1-t)f\bigr)+ \lambda \Omega\bigl(tf_{\mathbf{z}}+(1-t)f\bigr) \\ & \leq t\mathcal{E}_{\mathbf{z}}(f_{\mathbf{z}})+(1-t) \mathcal{E}_{\mathbf{z}}(f)+\lambda \biggl[t\Omega(f_{\mathbf{z}})+(1-t) \Omega(f)-\frac{\gamma}{2} t(1-t)\Vert f-f_{\mathbf{z}}\Vert _{K}^{2}\biggr]. \end{aligned}$$

This leads to

$$(1-t) \bigl(\mathcal{E}_{\mathbf{z}}(f_{\mathbf{z}})+\lambda \Omega(f_{\mathbf{z}})\bigr) \leq (1-t) \bigl(\mathcal{E}_{\mathbf{z}}(f)+\lambda \Omega(f)\bigr)-\frac{\lambda\gamma}{2} t(1-t) \Vert f-f_{\mathbf{z}}\Vert _{K}^{2}, $$

*i.e.*,

$$\mathcal{E}_{\mathbf{z}}(f_{\mathbf{z}})+\lambda \Omega(f_{\mathbf{z}}) \leq \mathcal{E}_{\mathbf{z}}(f)+\lambda \Omega(f)-\frac{\lambda\gamma}{2} t \Vert f-f_{\mathbf{z}}\Vert _{K}^{2}. $$

Let *t* tend to 1, we have

$$\mathcal{E}_{\mathbf{z}}(f_{\mathbf{z}})+\lambda \Omega(f_{\mathbf{z}}) \leq \mathcal{E}_{\mathbf{z}}(f)+\lambda \Omega(f)-\frac{\lambda\gamma}{2} \Vert f-f_{\mathbf{z}}\Vert _{K}^{2} $$

for any $f \in \mathcal{H}_{K}$. Similarly, we also have

$$\mathcal{E}_{\bar{\mathbf{z}}}(f_{\bar{\mathbf{z}}})+\lambda \Omega(f_{\bar{\mathbf{z}}}) \leq \mathcal{E}_{\mathbf{z}}(f)+\lambda \Omega(f)-\frac{\lambda\gamma}{2} \Vert f-f_{\bar{\mathbf{z}}}\Vert _{K}^{2} $$

for any $f \in \mathcal{H}_{K}$. Therefore,

$$\begin{aligned}& \mathcal{E}_{\mathbf{z}}(f_{\mathbf{z}})+\lambda \Omega(f_{\mathbf{z}}) \leq \mathcal{E}_{\mathbf{z}}(f_{\bar{\mathbf{z}}})+\lambda \Omega(f_{\bar{\mathbf{z}}})- \frac{\lambda\gamma}{2} \Vert f_{\bar{\mathbf{z}}}-f_{\mathbf{z}}\Vert _{K}^{2}, \\& \mathcal{E}_{\bar{\mathbf{z}}}(f_{\bar{\mathbf{z}}})+\lambda \Omega(f_{\bar{\mathbf{z}}}) \leq \mathcal{E}_{\bar{\mathbf{z}}}(f_{\bar{\mathbf{z}}})+\lambda \Omega(f_{\bar{\mathbf{z}}})- \frac{\lambda\gamma}{2} \Vert f_{\mathbf{z}}-f_{\bar{\mathbf{z}}}\Vert _{K}^{2}. \end{aligned}$$

By adding the two equations we have

$$\begin{aligned} \lambda \gamma \Vert f_{\bar{\mathbf{z}}}-f_{\mathbf{z}}\Vert _{K}^{2} &\leq \bigl( \mathcal{E}_{\bar{\mathbf{z}}}(f_{\mathbf{z}})- \mathcal{E}_{\mathbf{z}}(f_{\mathbf{z}}) \bigr)+ \bigl( \mathcal{E}_{\bar{\mathbf{z}}}(f_{\mathbf{z}}) \mathcal{E}_{\bar{\mathbf{z}}}(f_{\bar{\mathbf{z}}}) \bigr) \\ & =\frac{1}{m} \bigl( L\bigl(f_{\mathbf{z}}(\bar{x}_{m}), \bar{y}_{m}\bigr)-L\bigl(f_{\mathbf{z}}(x_{m}), y_{m}\bigr) \bigr)+\frac{1}{m} \bigl( L\bigl(f_{\bar{\mathbf{z}}}(x_{m}), y_{m}\bigr)-L\bigl(f_{\bar{\mathbf{z}}}(\bar{x}_{m}), \bar{y}_{m}\bigr) \bigr) \\ & \leq \frac{2C_{L}}{m} \Vert f_{\mathbf{z}}-f_{\bar{\mathbf{z}}}\Vert _{\infty}. \end{aligned}$$

From the fact that $\Vert f\Vert _{\infty}=\sup_{x \in X} \vert f(x)\vert \leq \sup_{x \in X} \langle f, K_{x} \rangle_{K} \leq \kappa \Vert f\Vert _{K}$ for any $f \in \mathcal{H}_{K}$ we have

$$\Vert f_{\bar{\mathbf{z}}}-f_{\mathbf{z}}\Vert _{K} \leq \frac{2\kappa C_{L}}{\lambda \gamma m}, $$

and the theorem is proved. □

Though the condition for the latter result is stronger than the first one, we will still apply this to the analysis below, as the bound is sharper and most of the loss functions satisfy the Lipschitz condition above.

## Differential private learning algorithms

In this section, we will describe the general differential private learning algorithms based on an output perturbation method. Perturbation ERM algorithms give a random output by adding a random perturbation term on the above deterministic output. That is,

2 $$ f_{\mathcal{A},{\mathbf{z}}}=f_{\mathbf{z}}+b, $$

where $f_{\mathbf{z}}$ is derived from (1). To determine the distribution of *b*, we firstly recall the sensitivity, introduced in Dwork 2006 [2], in our settings.

### Definition 2

We denote Δ*f* as the maximum infinite norm of difference between the outputs when changing one sample point in **z**. Let **z** and $\bar{\mathbf{z}}$ be defined as in the previous section, and $f_{\mathbf{z}}$ and $f_{\bar{\mathbf{z}}}$ be derived from (1) accordingly, we can see that

$$\Delta f:=\sup_{{\mathbf{z}}, \bar{\mathbf{z}}} \Vert f_{\mathbf{z}}-f_{\bar{\mathbf{z}}} \Vert _{\infty}. $$

Then a similar result to [2] is the following.

### Lemma 1

*Assume* Δ*f* *is bounded by* $B_{\Delta}>0$, *and* *b* *has a density function proportional to* $\exp\{-\frac{\epsilon \vert b\vert }{B_{\Delta}}\}$, *then algorithm* (2) *provides* *ϵ*-*differential privacy*.

### Proof

For all possible output function *r*, and **z**, ${\bar{\mathbf{z}}}$ differ in last element,

$$\Pr \{f_{{\mathbf{z}}, \mathcal{A}}=r\}=\Pr_{b} \{b=r-f_{\mathbf{z}}\} \propto \exp \biggl( -\frac{\epsilon \vert r-f_{\mathbf{z}}\vert }{B_{\Delta}} \biggr) $$

and

$$\Pr \{f_{\bar{\mathbf{z}}, \mathcal{A}}=r\}=\Pr_{b} \{b=r-f_{\bar{\mathbf{z}}}\} \propto \exp \biggl( -\frac{\epsilon \vert r-f_{\bar{\mathbf{z}}}\vert }{B_{\Delta}} \biggr). $$

So by the triangle inequality,

$$\Pr \{f_{{\mathbf{z}}, \mathcal{A}}=r\} \leq \Pr \{f_{\bar{\mathbf{z}}, \mathcal{A}}=r\} \times e^{\frac{\epsilon \vert f_{\mathbf{z}} -f_{\bar{\mathbf{z}}}\vert }{B_{\Delta}}} \leq e^{\epsilon}\Pr \{f_{\bar{\mathbf{z}}, \mathcal{A}}=r\}. $$

Then the lemma is proved by a union bound. □

Combining this with the result in the previous section, we can choose the noise term *b* as follows.

### Proposition 1

*Assume the conditions in Theorem* 1 *hold*, *and* *b* *takes value in* $(-\infty, +\infty)$, *we choose the density of* *b* *to be* $\frac{1}{\alpha}\exp ( -\frac{\lambda \gamma m \epsilon \vert b\vert }{\kappa^{2} C_{L}} )$, *where* $\alpha=\frac{2\kappa^{2} C_{L}}{\lambda \gamma m \epsilon}$, *then the algorithm* (2) *provides* *ϵ*-*differential privacy*.

### Proof

Since from the previous section we have

$$\Vert f_{\mathbf{z}}-f_{\bar{\mathbf{z}}}\Vert _{K} \leq \frac{2\kappa C_{L}}{\lambda \gamma m} $$

for any **z** and $\bar{\mathbf{z}}$ differing in the last sample point. Then from the reproducing property,

$$\Delta f_{\mathbf{z}}=\sup_{{\mathbf{z}},{\bar{\mathbf{z}}}} \Vert f_{\mathbf{z}}-f_{\bar{\mathbf{z}}}\Vert _{\infty}\leq \frac{2\kappa^{2} C_{L}}{\lambda \gamma m}. $$

The proposition is proved by substitute $B_{\Delta}=\frac{2\kappa^{2} C_{L}}{\lambda \gamma m}$ in the last lemma. □

## Error analysis

In this section, we conduct the error analysis for the general differential private ERM algorithm (2). We denote

$$f_{\rho}=\arg\min_{f} \mathcal{E}(f)=\arg\min _{f} \int_{Z} L\bigl(f(x),y\bigr) \,d\rho $$

as our goal function. In the following in this section, we always assume the Lipshitz continuous condition for the loss function, *i.e.* $\vert L(t_{1},y)-L(t_{2},y)\vert \leq C_{L} \vert t_{1}-t_{2}\vert $ for any $t_{1}$, $t_{2}$, *y* and some $C_{L}>0$. Now let us introduce our error decomposition,

3 $$ \begin{aligned}[b] \mathcal{E}(f_{{\mathbf{z}}, \mathcal{A}})- \mathcal{E}(f_{\rho}) &\leq \mathcal{E}(f_{{\mathbf{z}}, \mathcal{A}})- \mathcal{E}(f_{\rho}) +\lambda \Omega(f_{\mathbf{z}}) \\ & \leq \mathcal{E}(f_{{\mathbf{z}}, \mathcal{A}})-\mathcal{E}_{\mathbf{z}} (f_{{\mathbf{z}}, \mathcal{A}})+\mathcal{E}_{\mathbf{z}}(f_{{\mathbf{z}}, \mathcal{A}}) - \mathcal{E}_{\mathbf{z}}(f_{\mathbf{z}})+\mathcal{E}_{\mathbf{z}} (f_{\mathbf{z}})+\lambda \Omega(f_{\mathbf{z}}) -\mathcal{E}(f_{\rho}) \\ &\leq \mathcal{E}(f_{{\mathbf{z}}, \mathcal{A}})-\mathcal{E}_{\mathbf{z}} (f_{{\mathbf{z}}, \mathcal{A}})+\mathcal{E}_{\mathbf{z}}(f_{{\mathbf{z}}, \mathcal{A}}) - \mathcal{E}_{\mathbf{z}}(f_{\mathbf{z}})+\mathcal{E}_{\mathbf{z}} (f_{\lambda})+\lambda \Omega(f_{\lambda})-\mathcal{E}(f_{\rho}) \\ & \leq \mathcal{R}_{1} + \mathcal{R}_{2} + \mathcal{S} + D(\lambda), \end{aligned} $$

where $f_{\lambda}$ is a function in $\mathcal{H}_{K}$ to be determined and

$$\begin{aligned}& \mathcal{R}_{1}=\mathcal{E}(f_{{\mathbf{z}}, \mathcal{A}})-\mathcal{E}_{\mathbf{z}} (f_{{\mathbf{z}}, \mathcal{A}}),\qquad \mathcal{R}_{2}=\mathcal{E}_{\mathbf{z}}(f_{{\mathbf{z}}, \mathcal{A}}) -\mathcal{E}_{\mathbf{z}}(f_{\mathbf{z}}), \\& \mathcal{S}=\mathcal{E}_{\mathbf{z}}(f_{\lambda})- \mathcal{E}(f_{\lambda}),\qquad D(\lambda)=\mathcal{E}(f_{\lambda})-\mathcal{E}(f_{\rho})+\lambda \Omega(f_{\lambda}). \end{aligned}$$

Here $\mathcal{R}_{1}$ and $\mathcal{R}_{2}$ involve the function $f_{{\mathbf{z}}, \mathcal{A}}$ from random algorithm (2) so we call them random errors. $\mathcal{S}$ and $D(\lambda)$ are similar to the classical ones in the literature in learning theory and are called sample error and approximation error. In the following we will study these errors, respectively.

### Concentration inequality and error bounds for random errors

To bound the first random error, we need a concentration inequality. Dwork *et al.* 2015 [23] have proposed such an inequality under their differential private setting. Soon Bassily *et al.* 2015 [13] gave a different proof for the concentration inequality, which enlightens our error analysis.

#### Theorem 3

*If an algorithm* $\mathcal{A}$ *provides* *ϵ*-*differential privacy*, *and outputs a positive function* $g_{{\mathbf{z}}, \mathcal{A}}: Z \to \mathbb{R}$ *with bounded expectation* $\mathbb{E}_{{\mathbf{z}}, \mathcal{A}} \frac{1}{m} \sum_{i=1}^{m} g_{{\mathbf{z}}, \mathcal{A}}(z_{i}) \leq G$ *some* $G>0$, *where the expectation is taken over the sample and the output of the random algorithm*. *Then*

$$\mathbb{E}_{{\mathbf{z}}, \mathcal{A}} \Biggl( \frac{1}{m} \sum _{i=1}^{m} g_{{\mathbf{z}}, \mathcal{A}}(z_{i})- \int_{Z} g_{{\mathbf{z}}, \mathcal{A}}(z) \,d\rho \Biggr) \leq 2G\epsilon $$

*and*

$$\mathbb{E}_{{\mathbf{z}}, \mathcal{A}} \Biggl( \int_{Z} g_{{\mathbf{z}}, \mathcal{A}}(z) \,d\rho-\frac{1}{m} \sum _{i=1}^{m} g_{{\mathbf{z}}, \mathcal{A}}(z_{i}) \Biggr) \leq 2G\epsilon. $$

#### Proof

Denote the sample sets ${\mathbf{w}}_{j}=\{z_{1}, z_{2}, \ldots, z_{j-1}, z_{j}', z_{j+1}, \ldots, z_{m}\}$ for $j \in \{1,2, \ldots, m\}$. We observe that

$$\begin{aligned}& \mathbb{E}_{{\mathbf{z}}, \mathcal{A}} \Biggl( \frac{1}{m} \sum _{i=1}^{m} g_{{\mathbf{z}}, \mathcal{A}}(z_{i}) \Biggr)\\& \quad = \frac{1}{m} \sum_{i=1}^{m} \mathbb{E}_{\mathbf{z}} \mathbb{E}_{\mathcal{A}} \bigl( g_{{\mathbf{z}}, \mathcal{A}}(z_{i}) \bigr) \\& \quad =\frac{1}{m} \sum_{i=1}^{m} \mathbb{E}_{\mathbf{z}} \mathbb{E}_{z_{i}'} \int_{0}^{+\infty} \Pr_{\mathcal{A}} \bigl\{ g_{{\mathbf{z}}, \mathcal{A}}(z_{i}) \geq t \bigr\} \,dt \leq \frac{1}{m} \sum_{i=1}^{m} \mathbb{E}_{\mathbf{z}} \mathbb{E}_{z_{i}'} \int_{0}^{+\infty} e^{\epsilon}\Pr_{\mathcal{A}} \bigl\{ g_{{\mathbf{w}}_{i}, \mathcal{A}}(z_{i}) \geq t \bigr\} \,dt \\& \quad =e^{\epsilon}\frac{1}{m} \sum_{i=1}^{m} \mathbb{E}_{{\mathbf{w}}_{i}} \mathbb{E}_{z_{i}} \mathbb{E}_{\mathcal{A}} \bigl( g_{{\mathbf{w}}_{i}, \mathcal{A}}(z_{i}) \bigr) =e^{\epsilon}\frac{1}{m} \sum_{i=1}^{m} \mathbb{E}_{{\mathbf{w}}_{i}, \mathcal{A}} \mathbb{E}_{z_{i}} \bigl( g_{{\mathbf{w}}_{i}, \mathcal{A}}(z_{i}) \bigr) \\& \quad =e^{\epsilon}\frac{1}{m} \sum_{i=1}^{m} \mathbb{E}_{{\mathbf{w}}_{i}, \mathcal{A}} \int_{Z} g_{{\mathbf{w}}_{i}, \mathcal{A}}(z) \,d\rho =e^{\epsilon}\frac{1}{m} \sum_{i=1}^{m} \mathbb{E}_{{\mathbf{z}}, \mathcal{A}} \int_{Z} g_{{\mathbf{z}}, \mathcal{A}}(z) \,d\rho \\& \quad =e^{\epsilon}\mathbb{E}_{{\mathbf{z}}, \mathcal{A}} \int_{Z} g_{{\mathbf{z}}, \mathcal{A}}(z) \,d\rho. \end{aligned}$$

Then

$$\begin{aligned}& \mathbb{E}_{{\mathbf{z}}, \mathcal{A}} \Biggl( \frac{1}{m} \sum _{i=1}^{m} g_{{\mathbf{z}}, \mathcal{A}}(z_{i})- \int_{Z} g_{{\mathbf{z}}, \mathcal{A}}(z) \,d\rho \Biggr) \\& \quad \leq \bigl(1-e^{-\epsilon}\bigr) \mathbb{E}_{{\mathbf{z}}, \mathcal{A}} \Biggl( \frac{1}{m} \sum_{i=1}^{m} g_{{\mathbf{z}}, \mathcal{A}}(z_{i}) \Biggr) \leq 2G\epsilon. \end{aligned}$$

On the other hand,

$$\begin{aligned}& \mathbb{E}_{{\mathbf{z}}, \mathcal{A}} \int_{Z} g_{{\mathbf{z}}, \mathcal{A}} (z) \,d\rho\\& \quad =\frac{1}{m} \sum _{i=1}^{m} \mathbb{E}_{\mathbf{z}} \mathbb{E}_{\mathcal{A}} \int_{Z} g_{{\mathbf{z}}, \mathcal{A}}(z) \,d\rho \\& \quad =\frac{1}{m} \sum_{i=1}^{m} \mathbb{E}_{{\mathbf{w}}_{i}} \mathbb{E}_{\mathcal{A}} \int_{Z} g_{{\mathbf{w}}_{i}, \mathcal{A}}(z) \,d\rho=\frac{1}{m} \sum _{i=1}^{m} \mathbb{E}_{{\mathbf{w}}_{i}} \mathbb{E}_{\mathcal{A}} \int_{Z} g_{{\mathbf{w}}_{i}, \mathcal{A}}(z_{i}) \,d \rho(z_{i}) \\& \quad =\frac{1}{m} \sum_{i=1}^{m} \mathbb{E}_{{\mathbf{w}}_{i}} \mathbb{E}_{z_{i}} \mathbb{E}_{\mathcal{A}} \bigl( g_{{\mathbf{w}}_{i}, \mathcal{A}}(z_{i}) \bigr)=\frac{1}{m} \sum _{i=1}^{m} \mathbb{E}_{\mathbf{z}} \mathbb{E}_{z_{i}'} \int_{0}^{+\infty} \Pr_{\mathcal{A}} \bigl\{ g_{{\mathbf{w}}_{i}, \mathcal{A}}(z_{i}) \geq t \bigr\} \,dt \\& \quad \leq \frac{1}{m} \sum_{i=1}^{m} \mathbb{E}_{\mathbf{z}} \mathbb{E}_{z_{i}'} e^{\epsilon}\int_{0}^{+\infty} \Pr_{\mathcal{A}} \bigl\{ g_{{\mathbf{z}}, \mathcal{A}}(z_{i}) \geq t \bigr\} \,dt \\& \quad =e^{\epsilon}\frac{1}{m} \sum_{i=1}^{m} \mathbb{E}_{\mathbf{z}} \mathbb{E}_{\mathcal{A}} \bigl( g_{{\mathbf{z}}, \mathcal{A}}(z_{i}) \bigr)=e^{\epsilon}\mathbb{E}_{{\mathbf{z}}, \mathcal{A}} \frac{1}{m} \sum_{i=1}^{m} g_{{\mathbf{z}}, \mathcal{A}}(z_{i}). \end{aligned}$$

This leads to

$$\begin{gathered} \mathbb{E}_{{\mathbf{z}}, \mathcal{A}} \Biggl( \int_{Z} g_{{\mathbf{z}}, \mathcal{A}}(z) \,d\rho-\frac{1}{m} \sum _{i=1}^{m} g_{{\mathbf{z}}, \mathcal{A}}(z_{i}) \Biggr) \\ \quad =\bigl(e^{\epsilon}-1\bigr) \mathbb{E}_{{\mathbf{z}}, \mathcal{A}} \frac{1}{m} \sum_{i=1}^{m} g_{{\mathbf{z}}, \mathcal{A}}(z_{j}) \leq 2G\epsilon. \end{gathered} $$

These verify our results. □

#### Remark 1

In [23] and [13], the authors restrict the function to take values in $[0,1]$ or $\{0,1\}$ for their special use, our result here extends the result to the function taking values in $\mathbb{R}^{+}$. This makes our following error analysis implementable.

Since *y* is bounded by $M>0$ throughout our paper, it is reasonable to assume that $\mathcal{E}_{\mathbf{z}}(0)=\frac{1}{m} \sum_{i=1}^{m} L(0,y_{i}) \leq B_{0}$ for some $B_{0}>0$ depending just on *M*. Then we apply this concentration inequality to the random error $\mathcal{R}_{1}$.

#### Proposition 2

*Let* $f_{{\mathbf{z}}, \mathcal{A}}$ *be obtained from algorithm* (2). *Assume* $\mathcal{E}_{\mathbf{z}}(0) \leq B_{0}$ *for some constant* $B_{0}>0$. *We have*

$$\mathbb{E}_{{\mathbf{z}},\mathcal{A}} \mathcal{R}_{1}=\mathbb{E}_{{\mathbf{z}},\mathcal{A}} \bigl( \mathcal{E}(f_{{\mathbf{z}}, \mathcal{A}})-\mathcal{E}_{\mathbf{z}} (f_{{\mathbf{z}}, \mathcal{A}}) \bigr) \leq 2\tilde{B}\epsilon+2\epsilon\mathbb{E}_{{\mathbf{z}},\mathcal{A}} \mathcal{R}_{2}, $$

*where* $\tilde{B}=2(B_{0}+\lambda \Omega(0))$ *is a constant independent of* *m*.

#### Proof

Let $g_{{\mathbf{z}}, \mathcal{A}}(z)=L(f_{{\mathbf{z}}, \mathcal{A}}(x),y)$, which is always positive. Note that

$$\mathbb{E}_{{\mathbf{z}},\mathcal{A}} \Biggl( \frac{1}{m} \sum _{i=1}^{m} g_{{\mathbf{z}}, \mathcal{A}}(z_{i}) \Biggr)= \frac{1}{m} \sum_{i=1}^{m} \mathbb{E}_{{\mathbf{z}},\mathcal{A}} L\bigl(f_{{\mathbf{z}}, \mathcal{A}}(x_{i}),y_{i} \bigr)=\mathbb{E}_{{\mathbf{z}},\mathcal{A}} \mathcal{R}_{2}+ \mathbb{E}_{{\mathbf{z}},\mathcal{A}} \mathcal{E}_{\mathbf{z}}(f_{\mathbf{z}}) $$

and

$$\mathcal{E}_{\mathbf{z}}(f_{\mathbf{z}}) \leq \mathcal{E}_{\mathbf{z}}(f_{\mathbf{z}})+ \lambda \Omega(f_{\mathbf{z}}) \leq \mathcal{E}_{\mathbf{z}}(0)+\lambda \Omega(0) \leq B_{0}+\lambda \Omega(0), $$

we have

$$\mathbb{E}_{{\mathbf{z}},\mathcal{A}} \Biggl( \frac{1}{m} \sum _{i=1}^{m} g_{{\mathbf{z}}, \mathcal{A}}(z_{i}) \Biggr) \leq \mathbb{E}_{{\mathbf{z}},\mathcal{A}} \mathcal{R}_{2}+B_{0}+\lambda \Omega(0). $$

By applying the concentration inequality for the given $g_{{\mathbf{z}}, \mathcal{A}}$ we can prove the result with constant $\tilde{B}=2(B_{0}+\lambda \Omega(0))$. □

For the random error $\mathcal{R}_{2}$, we have the following estimation.

#### Proposition 3

*For the function* $f_{{\mathbf{z}}, \mathcal{A}}$ *obtained from algorithm* (2), *we have*

$$\mathbb{E}_{{\mathbf{z}},\mathcal{A}} \mathcal{R}_{2}=\mathbb{E}_{{\mathbf{z}},\mathcal{A}} \bigl( \mathcal{E}_{\mathbf{z}}(f_{{\mathbf{z}}, \mathcal{A}}) -\mathcal{E}_{\mathbf{z}}(f_{\mathbf{z}}) \bigr) \leq \frac{\kappa^{2} C_{L}}{\lambda \gamma m \epsilon}. $$

#### Proof

Note that

$$\bigl\vert L\bigl(f_{{\mathbf{z}},\mathcal{A}}(x_{i}),y_{i}\bigr)-L \bigl(f_{\mathbf{z}}(x_{i}),y_{i}\bigr)\bigr\vert \leq C_{L} \bigl\vert f_{{\mathbf{z}},\mathcal{A}}(x_{i})-f_{\mathbf{z}}(x_{i}) \bigr\vert =C_{L} \vert b\vert . $$

Therefore,

$$\begin{aligned} \mathbb{E}_{{\mathbf{z}},\mathcal{A}} \mathcal{R}_{2}&=\mathbb{E}_{{\mathbf{z}},\mathcal{A}} \Biggl( \frac{1}{m} \sum_{i=1}^{m} \bigl[L\bigl(f_{{\mathbf{z}},\mathcal{A}}(x_{i}),y_{i}\bigr) -L \bigl(f_{\mathbf{z}}(x_{i}),y_{i}\bigr)\bigr] \Biggr) \\ & \leq \mathbb{E}_{{\mathbf{z}},\mathcal{A}} C_{L} \vert b\vert =C_{L} \mathbb{E}_{b} \vert b\vert =\frac{\kappa^{2} C_{L}}{\lambda \gamma m \epsilon}. \end{aligned}$$

This verifies our bound. □

### Error estimate for the other error terms

For the sample error and approximation error, we choose $f_{\lambda}$ to be some function in $\mathcal{H}_{K}$ close to $f_{\rho}$, which satisfies $\vert L(f_{\lambda}(x),y)\vert \leq B_{\rho}$ for some $B_{\rho}>0$. Explicit expressions of $f_{\lambda}$ and $B_{\rho}$ will be presented in the next section, with respect to different algorithms. To bound the sample error, we should recall the Hoeffding inequality [24].

#### Lemma 2

*Let* *ξ* *be a random variable on a probability space* *Z* *satisfying* $\vert \xi(z)-\mathbb{E}\xi \vert \leq \Xi$ *for some* $\Xi>0$ *for almost all* $z \in Z$. *Denote* $\sigma^{2}=\sigma^{2}(\xi)$, *then*, *for any* $t>0$,

$$\Pr \Biggl\{ \Biggl\vert \frac{1}{m} \sum_{i=1}^{m} \xi(z_{i})-\mathbb{E}\xi \ge t \Biggr\vert \Biggr\} \leq 2 \exp \biggl\{ -\frac{m t^{2}}{2\Xi^{2}} \biggr\} . $$

Now we have the following proposition.

#### Proposition 4

*Let* $L(f_{\lambda}(x),y) \leq B_{\rho}$ *for any* $(x,y) \in Z$, *we have*

$$\mathbb{E}_{{\mathbf{z}},\mathcal{A}} \mathcal{S} \leq \frac{2\sqrt{2\pi}B_{\rho}}{\sqrt{m}}. $$

#### Proof

Since

$$\mathcal{S}= \int_{Z} L\bigl(f_{\lambda}(x),y\bigr) \,d\rho- \frac{1}{m} \sum_{i=1}^{m} L \bigl(f_{\lambda}(x_{i}),y_{i}\bigr), $$

we apply the Hoeffding inequality to $\xi(z)=-L(f_{\lambda}(x),y)$. Note that $\vert \xi-\mathbb{E}\xi \vert \leq 2B_{\rho}$ and

$$\Pr_{\mathbf{z}} \Biggl\{ \Biggl\vert \int_{Z} L\bigl(f_{\lambda}(x),y\bigr) \,d\rho- \frac{1}{m} \sum_{i=1}^{m} L \bigl(f_{\lambda}(x_{i}),y_{i}\bigr) \Biggr\vert \ge \varepsilon \Biggr\} \leq 2 \exp \biggl\{ -\frac{m\varepsilon^{2}}{8B_{\rho}^{2}} \biggr\} . $$

Therefore

$$\begin{aligned} \mathbb{E}_{{\mathbf{z}}, \mathcal{A}} \mathcal{S} &\leq \mathbb{E}_{\mathbf{z}} \vert \mathcal{S}\vert = \int_{0}^{+\infty} \Pr_{\mathbf{z}} \bigl\{ \vert \mathcal{S}\vert \ge t\bigr\} \,dt \\ & \leq \int_{0}^{+\infty} 2\exp \biggl\{ -\frac{mt^{2}}{8B_{\rho}^{2}} \biggr\} \,dt \leq \frac{2\sqrt{2\pi}B_{\rho}}{\sqrt{m}}, \end{aligned}$$

and the proposition is proved. □

Let us turn to the approximation error $D(\lambda)$. It is difficult to give the upper bound for the abstract approximation error. So we use the natural assumption on $D(\lambda)$, which is

4 $$ D(\lambda) \leq c_{\beta}\lambda^{\beta}, $$

for some $0<\beta<1$ and $c_{\beta}>0$. This assumption is trivial in concrete algorithms; see [25–27], etc.

### Total error bound

Now we can deduce our total error by combining all the error bounds above.

#### Theorem 4

*Let* $f_{{\mathbf{z}},\mathcal{A}}$ *defined as* (2), $f_{\rho}$ *defined as above*. *Assume* $\mathcal{E}_{\mathbf{z}}(0) \leq B_{0}$, $\vert L(f_{\lambda}(x),y)\vert \leq B_{\rho}$, *and* (4) *hold*. *By choosing* $\epsilon=1/\sqrt{\lambda m}$ *and* $\lambda=m^{-1/(2\beta+1)}$ *we have*

$$\mathbb{E}_{{\mathbf{z}},\mathcal{A}} \bigl( \mathcal{E}(f_{{\mathbf{z}},\mathcal{A}}) - \mathcal{E}(f_{\rho}) \bigr) \leq \biggl( 2B_{0}+2 \Omega(0) + \frac{3\kappa^{2}C_{L}}{\gamma} +c_{\beta}\biggr) \biggl( \frac{1}{m} \biggr)^{\frac{\beta}{2\beta+1}}. $$

#### Proof

By substituting the upper bounds above in the error decomposition (3), we have

$$\mathbb{E}_{{\mathbf{z}},\mathcal{A}} \bigl( \mathcal{E}(f_{{\mathbf{z}},\mathcal{A}}) - \mathcal{E}(f_{\rho}) \bigr) \leq 2\bigl(B_{0}+\lambda \Omega(0)\bigr) \epsilon+ \frac{(1+2\epsilon)\kappa^{2}C_{L}}{\lambda\gamma m \epsilon} +\frac{2\sqrt{2\pi}B_{\rho}}{\sqrt{m}} +c_{\beta}\lambda^{\beta}. $$

Take $\epsilon=1/\sqrt{\lambda m}$ and $\lambda=m^{-1/(2\beta+1)}$ for balance, then the result is proved. □

Here we present a general convergence result for the general differential private ERM learning algorithms. In this theorem, we provide a choice for the parameters *ϵ* and *λ*, under some conditions above, which leads to a learning rate $m^{-\beta/(2\beta+1)}$ with fixed *B* and *γ*. However, in an explicit algorithm *B* and *γ* may depend on *λ* and the learning rate will vary accordingly. We cannot go further without a specific description of the algorithms, which will be studied in the next section.

## Applications

In this section, we will apply our results to several frequently used learning algorithms. First of all, let us take a look at the assumptions as regards $f_{\rho}$. Denote the integral operator $L_{K}$ as $L_{K}f(t)=\int_{X} f(x)K(x,t) \,d\rho_{X}(x)$. It is well known that [18] $\Vert L_{K}\Vert \leq \kappa^{2}$. Then $f_{\rho}\in L_{K}^{r}(L_{\rho_{X}}^{2})$ for some $r>0$ is often used in learning theory literature. When $r=1/2$, it is the same as $f_{\rho}\in \mathcal{H}_{K}$ [18]. It is natural if we consider $L(\pi(f(x)),y) \leq L(f(x),y)$ for any function *f* and $(x,y) \in Z$, which means $\pi(f(x))$ is more close than $f(x)$ to *y* in some sense, as $\vert y\vert \leq M$. Here

$$\pi\bigl(f(x)\bigr)= \textstyle\begin{cases} M, & f(x)>M, \\ f(x), & -M \leq f(x) \leq M ,\\ -M, & f(x)< -M. \end{cases} $$

Then $\int_{Z} (\pi(f_{\rho}(x)),y) \,d\rho \leq \int_{Z} (f_{\rho}(x),y) \,d\rho$, *i.e.*, $\vert f_{\rho}(x)\vert \leq M$ always holds. So without loss of generality, we also assume $\Vert f_{\rho} \Vert _{\infty}\leq M$.

### Differential private least squares regularization

Our first example is the differential private least squares regularization algorithm,

$$f_{\mathbf{z}}^{ls}=\arg \min_{f \in \mathcal{H}_{K}} \frac{1}{m} \sum_{i=1}^{m} \bigl(f(x_{i}) -y_{i}\bigr)^{2}+\lambda \Vert f \Vert _{K}^{2}, $$

and perturbation

$$f_{{\mathbf{z}},\mathcal{A}}^{ls}=f_{\mathbf{z}}^{ls}+b_{ls}. $$

Such an algorithm has been studied in our previous work [28]. Now we will try to apply the above analysis. Firstly we can verify that $\Omega(f)=\Vert f\Vert _{K}^{2}$ is 2-strongly convex, *i.e.*, $\gamma=2$ in our settings. Since $\mathcal{E}_{\mathbf{z}}(f_{\mathbf{z}}^{ls})+\lambda \Vert f_{\mathbf{z}}^{ls}\Vert _{K}^{2} \leq \mathcal{E}_{\mathbf{z}}(0)+0 \leq M^{2}$ with $\vert y\vert \leq M$, we have $\Vert f_{\mathbf{z}}^{ls}\Vert _{K} \leq \frac{M}{\sqrt{\lambda}}$, which leads to $\Vert f_{\mathbf{z}}^{ls}\Vert _{\infty}\leq \frac{\kappa M}{\sqrt{\lambda}}$ for any ${\mathbf{z}} \in Z^{m}$. Therefore though the least square loss is not Lipschitz continuous, it satisfies

$$\begin{aligned}& \bigl\vert L\bigl(f_{S_{1}}^{ls}(x),y\bigr)-L \bigl(f_{S_{2}}^{ls}(x),y\bigr)\bigr\vert \\& \quad =\bigl\vert \bigl(f_{S_{1}}^{ls}(x)-y\bigr)^{2} - \bigl(f_{S_{2}}^{ls}(x)-y\bigr)^{2}\bigr\vert \\& \quad \leq \bigl\vert f_{S_{1}}^{ls}(x)+f_{S_{2}}^{ls}(x)-2y \bigr\vert \cdot \bigl\vert f_{S_{1}}^{ls}(x)-f_{S_{2}}^{ls}(x) \bigr\vert \leq \frac{2M(\kappa+1)}{\sqrt{\lambda}} \cdot \bigl\vert f_{S_{1}}^{ls}(x)-f_{S_{2}}^{ls}(x) \bigr\vert \end{aligned}$$

for any $S_{1}, S_{2} \in Z^{m}$. So we set $C_{L}=\frac{2M(\kappa+1)}{\sqrt{\lambda}}$ in Proposition 1. Then $b_{ls}$ has a density function $\frac{1}{\alpha}\exp \{-\frac{2\vert b\vert }{\alpha}\}$ with $\alpha=\frac{2M\kappa^{2}(\kappa+1)}{\lambda^{3/2} m \epsilon}$, which makes the algorithm provide *ϵ*-differential privacy.

A generalization analysis for this algorithm can also be found in [28]. What we shall mention here is that direct use of our error bound in the previous section leads to an unsatisfactory learning rate, since $C_{L}$ tends to ∞ when $m \to \infty$. However, note that

$$\bigl(f_{{\mathbf{z}},\mathcal{A}}^{ls}(x_{i})-y_{i} \bigr)^{2}-\bigl(f_{ls}(x_{i})-y_{i} \bigr)^{2}= 2b\bigl(f_{\mathbf{z}}^{ls}(x_{i})-y_{i} \bigr)+b^{2} $$

for any $i=1,2,\ldots,m$, then

$$\mathbb{E}_{{\mathbf{z}},\mathcal{A}} \bigl(\mathcal{E}_{\mathbf{z}}\bigl(f_{{\mathbf{z}}, \mathcal{A}}^{ls} \bigr)-\mathcal{E}_{\mathbf{z}}\bigl(f_{\mathbf{z}}^{ls}\bigr) \bigr) =\mathbb{E}_{b} b^{2}= \frac{2M^{2}\kappa^{4}(\kappa+1)^{2}}{\lambda^{3} m^{2} \epsilon^{2}}. $$

When $f_{\rho}^{ls} \in L_{K}^{r}(L_{\rho_{X}}^{2})$, let $f_{\lambda}=(L_{K}+\lambda I)^{-1}L_{K} f_{\rho}$, we have $B_{\rho}=4M^{2}$, and (4) holds with $\beta=\min\{1, 2r\}$ in Theorem 4 [29]. Then by choosing $\epsilon=1/(\lambda m^{\frac{2}{3}})$ and $\lambda=(1/m)^{\frac{2}{3(\beta+1)}}$, we can derive an error bound in the form of

$$\mathbb{E}_{{\mathbf{z}},\mathcal{A}} \bigl( \mathcal{E}\bigl(f_{{\mathbf{z}}, \mathcal{A}}^{ls} \bigr)-\mathcal{E}\bigl(f_{\rho}^{ls}\bigr) \bigr) \leq \tilde{C} (1/m)^{\frac{2\beta}{3(\beta+1)}} $$

for some *C̃* independent with *m*, from the total error bound in the last section. We omit the detail complex analysis here.

### Differential private SVM

The second example is differential private SVM. We describe the SVM algorithm as in [19], *i.e.*, when $Y=\{-1,+1\}$,

$$f_{\mathbf{z}}^{h}=\arg \min_{f \in \mathcal{H}_{K}} \frac{1}{m} \sum_{i=1}^{m} \bigl(1-y_{i}f(x_{i})\bigr)_{+} +\lambda \Vert f\Vert _{K}^{2}, $$

and perturbation

$$f_{{\mathbf{z}},\mathcal{A}}^{h}=f_{\mathbf{z}}^{h}+b_{h}, $$

where the hinge loss $L_{h}(f(x),y)=(1-yf(x))_{+} =\max\{0, 1-yf(x)\}$ is used in the ERM setting. Then the output classifier is $\operatorname{sgn}(f_{{\mathbf{z}},\mathcal{A}}^{h})$.

Firstly we consider the differential privacy of this algorithm. Note that $\vert a_{+}-b_{+}\vert \leq \vert a-b\vert $ for any $a,b \in \mathbb{R,}$ by the discussion, we have

$$\bigl\vert L\bigl(f_{1}(x),y\bigr)-L\bigl(f_{2}(x),y \bigr)\bigr\vert =\bigl\vert \bigl(1-yf_{1}(x)\bigr)_{+} - \bigl(1-yf_{2}(x)\bigr)_{+}\bigr\vert \leq \bigl\vert f_{1}(x)-f_{2}(x)\bigr\vert . $$

Then $C_{L}=1$ and $\gamma=2$ in Proposition 1. Therefore $b_{h}$ here has a density function $1/\alpha \exp\{ -\frac{2\vert b\vert }{\alpha} \}$ with $\alpha=\frac{\kappa^{2}}{\lambda m \epsilon}$. In this case, we have, for any possible output set $\mathcal{O}$,

$$\Pr\bigl\{ f_{{\mathbf{z}},\mathcal{A}}^{h} \in \mathcal{O} \bigr\} \leq e^{\epsilon}\Pr\bigl\{ f_{{\bar{\mathbf{z}}},\mathcal{A}}^{h} \in \mathcal{O} \bigr\} , $$

where $\bar{\mathbf{z}}$ differs from **z** in one element. Then, for any possible classifier *g* defined on *X*,

$$\Pr_{\mathcal{A}} \bigl\{ \operatorname{sgn}\bigl(f_{{\mathbf{z}},\mathcal{A}}^{h} \bigr)=g \bigr\} \leq e^{\epsilon}\Pr_{\mathcal{A}} \bigl\{ \operatorname{sgn}\bigl(f_{{\bar{\mathbf{z}}},\mathcal{A}}^{h}\bigr)=g \bigr\} . $$

This verifies the *ϵ*-differential privacy of the algorithm.

Now let us turn to the error analysis. When hinge loss is applied in the ERM setting, Theorem 14 of [30] reveals the comparison theorem, that is, denote $R(f)=\Pr(y \neq f(x))=\int_{X} \Pr(y \neq f(x)\vert x) \,d\rho_{X}$, then

$$R(f)-R(f_{c}) \leq \sqrt{2\bigl(\mathcal{E}(f)-\mathcal{E} \bigl(f_{\rho}^{h}\bigr)\bigr)} $$

for any measurable function *f*. Here

$$f_{\rho}^{h}=\arg\min_{f} \int_{Z} \bigl(1-yf(x)\bigr)_{+} \,d\rho. $$

Assume $f_{\rho}^{h} \in L_{K}^{r}(L_{\rho_{X}}^{2})$, for some $r>0$ and $f_{c}$ is the Bayes classifier, *i.e.*,

$$f_{c}(x)= \textstyle\begin{cases} 1, & \Pr(y=1\vert x) \ge \Pr(y=-1\vert x), \\ -1, & \Pr(y=1\vert x) < \Pr(y=-1\vert x). \end{cases} $$

Then

$$\mathbb{E}_{{\mathbf{z}}, \mathcal{A}} \bigl( R\bigl(f_{{\mathbf{z}},\mathcal{A}}^{h}\bigr) -R(f_{c}) \bigr) \leq \sqrt{2 \mathbb{E}_{{\mathbf{z}}, \mathcal{A}} \bigl( \mathcal{E}\bigl(f_{{\mathbf{z}},\mathcal{A}}^{h}\bigr) -\mathcal{E} \bigl(f_{\rho}^{h}\bigr) \bigr)}. $$

Still we choose stepping-stone function $f_{\lambda}=(L_{K}+\lambda I)^{-1} L_{K} f_{\rho}^{h}$, which leads to $\Vert f_{\lambda} \Vert _{\infty}\leq M$ and $B_{\rho}=(M+1)^{2}$. Reference [31] shows that $D(\lambda) \leq \lambda^{\min\{r,1\}}$, so we can follow the choice for *ϵ* and *λ* in Theorem 4 with $\beta=\min\{r,1\}$ to get the learning rate as

$$\mathbb{E}_{{\mathbf{z}}, \mathcal{A}} \bigl( R\bigl(f_{{\mathbf{z}},\mathcal{A}}^{h}\bigr) -R(f_{c}) \bigr) \leq \tilde{C} \biggl( \frac{1}{m} \biggr)^{\frac{\beta}{2(2\beta+1)}}, $$

where *C̃* is a constant independent of *m*.

## Results and conclusions

In this paper, we present two results in the analysis of the differential private convex risk minimization algorithms.

The first one is the perturbation results for general convex risk minimization algorithms. We studied two cases of the general algorithms. The second one is applied in the following analysis, as it leads to a sharper upper bound of the error between two outputs differ in 1 sample point. However, the first one is more relaxed, without Lipschitz continuity of the loss function. Based on such perturbation results we obtain a choice for the random terms of the differential private algorithms, *i.e.*, Proposition 1. This gives us a theoretical and practical construction of differential private algorithms.

An error analysis is the second contribution of this paper. The analysis relies on the concentration inequality in the setting of differential privacy. After conducting a different error decomposition using the above concentration inequality, we provide an upper bound or learning rate of the expected generalization error. In this result we find a selection of the parameter of differential privacy *ϵ* and the regularization parameter *λ*, both of which depend on the sample size *m*. Since smaller *ϵ* always means more effective privacy protection, this indicates that generalized algorithms must not be too much privacy protected.

In [8], the authors proposed that the learning rate can be $\frac{1}{2}$ under the strong assumption on the loss function and with regularization term $\frac{1}{2}\Vert f\Vert _{K}^{2}$. However, the differential private parameter *ϵ* is fixed there. In this paper we obtain a learning rate $\frac{1}{3}$ with weak conditions on the loss function and $r\ge\frac{1}{2}$ when choosing appropriate parameters *ϵ* and *λ*. As we pointed out above, *ϵ* should not be too small to derive convergent algorithms. In fact, for a fixed *ϵ*, we as well can deduce a learning rate of $\frac{1}{2}$ (with a slightly different form); see [28] for a detailed analysis.
